# An improved method using adaptive smoothing for GNSS tomographic imaging of ionosphere

**DOI:** 10.1371/journal.pone.0250613

**Published:** 2021-05-07

**Authors:** Rushang Jia, Xumin Yu, Jianping Xing, Yafei Ning, Hecheng Sun

**Affiliations:** 1 School of Microelectronics, Shandong University, Jinan, China; 2 Shanghai Key Laboratory of Space Navigation and Position Techniques, Shanghai, China; 3 National Key Lab. of Science and Technology on Space Microwave China Academy of Space Technology Xi’an, Xi’an, China; Universiti Sains Malaysia, MALAYSIA

## Abstract

Global navigation satellite system (GNSS) is a well-established sensors in the recent ionosphere research. By comparing with classical meteorological equipments, the GNSS application can obtain more reliable and precious ionospheric total electron content (TEC) result. However, the most used GNSS ionospheric tomography technique is sensitive to a priori information due to the sparse and non-uniform distribution of GNSS stations. In this paper, we propose an improved method based on adaptive Laplacian smoothing and algebraic reconstruction technique (ALS-ART). Compared with traditional constant constraints, this method is less dependent on a priori information and adaptive smoothing constraints is closer to the actual situation. Tomography experiments using simulated data show that reconstruction accuracy of ionospheric electron density using ALS-ART method is significantly improved. We also use the method to do the analysis of real observation data and compare the tomography results with ionosonde observation data. The results demonstrate the superiority and reliability of the proposed method compared to traditional constant constraints method which will further improve the capability of obtaining precious ionosphere TEC by using GNSS.

## 1. Introduction

The ionosphere is an ionized region of the Earth’s atmosphere, which is generally accepted that begins at 50 km and ends at 1000 km approximately from the Earth surface. In order to convert accurate integral measurements into 2-D or 3-D structures, a technique called tomography has been invented. In the field of global navigation satellite system (GNSS) atmosphere, the principle of computerized ionospheric tomography (CIT) becomes applicable with the increasing number of GNSS satellites and the build-up of ground-based GNSS stations in the 1990s [1–4]. Since then, a variety of CIT approaches based on GNSS observation data have been developed for the accurate reconstruction of ionospheric electron density (IED) distribution in the upper atmosphere [5–14]. An overview about the direction and challenges of an area at the forefront of CIT research is provided by Bust and Mitchell [15]. CIT is a typically inverse problem which is ill-posed with the limited number of viewing angles of the raypath measurement. To get the uniqueness of the CIT solution, there are many approaches which are used to solve the reconstruction sparseness.

Smoothing constraints approach can effectively constrain near voxels with no raypath information. Garcia and Crespon [16] applied local basis parametrisation of electron density which constrained by NeQuick ionosphere model and its spatial gradients. Hobiger et al. [17] used constrained simultaneous algebraic reconstruction technique for tomography reconstruction of electron density distribution and found that the convergence speed would be significantly higher than that of classical method. Lee and Kamalabadi [18] regularized the inverse problem by incorporating neighborhood smoothness and continuity constraints applicable to general ionospheric conditions. Wen et al. [19, 20] proposed constrained algebraic reconstruction technique for tomographic reconstruction and demonstrated the reliability and superiority of this method. Nesterov and Kunitsyn [21] developed minimal Sobolev’s norm constraint to smooth the solution. Yao et al. [22] incorporated Tikhonov and total variation regularization constraint to overcome near voxels with no raypath information. Panicciari et al. [23] permited sparsity in the inversion coefficient by using minimization constraint and wavelet basis functions. Norberg et al. [24, 25] used Bayesian statistical inversion with prior distribution given by its mean and covariance with ionosonde measurements and Gaussian Markov random fields as a sparse matrix for the numerical computations, and employed the required additional information with Bayesian which could be given with prior probability distributions, respectively. Wang et al. [26, 27] used model function in regularization constraint to balance the weights between observations and electron density field, and model function in the modified L-curve method to balance a prior information and real measurements, respectively. To a certain extent, these existing studies have overcome the ill-posedness of ionospheric electron density reconstruction based on constraints and priori information, especially in vertical structure of the ionosphere. These studies have shown that the results of CIT could strongly depend on constraints and prior information.

However, if the reconstruction relies excessively on a priori information in the CIT process, the tomographic results are similar to the prior information, and the CIT technology will lose its inherent value. To reduce the dependence on the prior information and reconstruct a higher precision ionospheric vertical structure, we propose a novel approach using adaptive Laplacian smoothing and algebraic reconstruction technique (ALS-ART) for three-dimensional GNSS ionospheric tomography. In the following, a more detailed discussion of CIT constraints is provided. Therefore, Section 2 describes the models for GNSS measurement and the principles of ionospheric tomography. Section 3 introduces how the adaptive smoothing is used for ionospheric electron density (IED). In Section 4, the proposed approach is applied to simulated and real tomography and this section also discusses the problems of CIT. Section 5 concludes the major findings.

## 2. Ionospheric tomography formulation

### 2.1 Ionospheric total electron content extraction

Ionospheric total electron content (TEC) is the integrated ionospheric electron density along the ray path between GNSS satellites and receivers. The TEC is commonly extracted from dual frequency GNSS receivers through methods of carrier phase smoothed pseudorange [28, 29] and uncombined precise point positioning (UPPP) [30, 31]. The TEC estimated by the method of carrier phase smoothed pseudorange is affected by code-delay multi-path and length of every continuous arc [32, 33]. Zhang et al. [30] proposed the UPPP method for estimating ionospheric observables, and they found that it effectively reduces leveling errors. The detailed introduction of the method can be found in Zhang et al. [30]. In this paper, we use the method to extract ionospheric TEC. The observation equations of the GNSS pseudorange and carrier phase can be generally expressed as

$$\{\begin{array}{c} P_{r,f}^{s}=\rho_{r}^{s}+c(\delta t_{r}-\delta t^{s})+T_{trop}+{\left(\frac{\lambda_{f}}{\lambda_{1}}\right)}^{2}I_{ion,1}+b_{r,f}^{s}+\varepsilon_{P}\\ L_{r,f}^{s}=\rho_{r}^{s}+c(\delta t_{r}-\delta t^{s})+T_{trop}-{\left(\frac{\lambda_{f}}{\lambda_{1}}\right)}^{2}I_{ion,1}+d_{r,f}^{s}-\lambda_{f}N_{r,f}^{s}+\varepsilon_{L} \end{array}$$ (1)

where $P_{r,f}^{s}$ and $L_{r,f}^{s}$ are pseudorange and carrier phase observation from receiver *r* to satellite *s* on frequency *f*(*f* = 1,2) in length, respectively; *ρ* is the geometry distance between the receiver and satellite; *c* is the speed of light; *δt*_*γ*_ and *δt*^*s*^ are the receiver and satellite clock offset, respectively; *T*_*trop*_ and *I*_*ion*_ are the tropospheric and ionospheric delay; *λ* is the wavelength; *b* is the frequency dependent code bias and *d* is the frequency dependent phase bias; *N* is the integer phase ambiguity in cycles; *ε* is the observation noise.

### 2.2 Tomography model

The slant TEC data are used as CIT projections in a region [34], which along the ray path of the GNSS signal between a satellite and a ground receiver is defined as the integrated value of the IED and is modeled as

$$\text{STEC}(t)=\int_{i}^{j}\text{Ne}(r,t)ds\;(i=1,\cdots ,I;\;j=1,\cdots ,J)$$ (2)

where STEC(*t*) is the slant TEC; Ne(*r*,*t*) is the electron density at the time *t*; *I* and *J* are the respective total number of receivers and satellites; and *i* and *j* are the positions of the *i*th ground receiver and *j*th satellite, respectively.

In a CIT approach, the ionosphere is divided into a grid in which each voxel has the same size or is not equidistant from other voxels. The linear integral in Eq (2) includes the slant TEC contribution along the entire ray path from the satellite to the receiver [18–22]. A parametric representation of Eq (2) can be written as

$$\text{STEC}(t)\approx \sum_{n=1}^{N}a_{n}\text{Ne}(r,t)+\varepsilon$$ (3)

where *n* and *a* denote a sampling point and the weights for numerical integration at the sampling points, respectively; *N* is the total number of voxels in the ionosphere grid; and *ε* is the measurement and model error.

The formulation in Eq (3) can be expressed as a discrete mathematical problem written as

Y=AX+ε (4)

where Y ∈ *R*^*M*×1^ is the slant TEC from GNSS observation values, *M* is the total number of slant TEC measurements, A ∈ *R*^*M*×*N*^ is the observation matrix that corresponds to the grid, X ∈ *R*^*N*×1^ is the IED at each voxel, and ε denotes the noise.

## 3. Tomography algorithm

### 3.1 Classic method and problem

Algebraic reconstruction technique (ART) is a row-action technique which is a classical iterative algorithm. It sets a priori information for each voxel before the iteration begins in the tomographic region. The *k*th iteration of ART algorithm computes the difference between Y and Y^*k*^ which is obtain by using the current estimated value X^*k*^ in Eq (4) [35–37]. A correction derived from the difference is distributed over X^*k*^ to get X^*k*+1^. After numbers of iterations, the tomography result converges to a solution of Eq (4). For the *k*th iteration, the ART algorithm can be written as

$${\text{X}}^{k+1}={\text{X}}^{k}+\gamma_{k}\frac{{\text{Y}}_{i}-\langle {\text{A}}_{i},{\text{X}}^{k}\rangle }{{\text{A}}_{i}{\text{A}}_{i}^{\text{T}}}{\text{A}}_{i}^{\text{T}}$$ (5)

where A_*i*_ is the *i*th row of matrix A. *k* is the number of iterations. *γ*_*k*_ is relaxation parameters which is usually chosen to be the same and confined to the interval (0,2).

Generally, the number of rays in tomography is larger than the number of voxels, so the ray geometry defines a non-singular matrix, which permits reconstruction of the detected media [17]. However, this is not usually the case in geoscience applications. For the ionospheric tomography, there are near voxels with no raypath information and the final values are often the same as a priori information. For this problem, additional constraints or a priori information is generally used for ionospheric tomography [17, 19]. The usual way to constrain the inverse problem is through damping and smoothing once it is linearized in Eq (4). A voxel without any ray passing through will extract information from their near voxels, while an over-determined voxel will determine their values through the data and contribute to the determination of their near voxels. These constraints can be written as

$$\{\begin{array}{c} 0=\text{HX}\\ 0=\text{VX}\\ 0=\text{BX} \end{array}$$ (6)

where H, V, B are the horizontal constraints, vertical constraints and boundary conditions. According to Eqs (4) and (6), we can get

$$\left(\begin{array}{c} \text{Y}\\ 0\\ 0\\ 0 \end{array})=(\begin{array}{c} \text{A}\\ \text{H}\\ \text{V}\\ \text{B} \end{array})\text{X+}(\begin{array}{c} \varepsilon \\ 0\\ 0\\ 0 \end{array}\right)$$ (7)

The Eq (7) can be expressed as

Y=SX+ε (8)

where S includes A, H, V and B. From Eqs (5) and (8), the algorithm combined by smoothing and ART can be given as

$${\text{X}}^{k+1}={\text{X}}^{k}+\gamma_{k}\frac{{\text{Y}}_{i}-\langle {\text{S}}_{i},{\text{X}}^{k}\rangle }{{\text{S}}_{i}{\text{S}}_{i}^{\text{T}}}{\text{S}}_{i}^{\text{T}}$$ (9)

The application of smoothing constraints indirectly increases the amount of observation information, which is equivalent to adding virtual observations to the voxels without any observation information, overcoming the problem of excessive dependence on a prior information.

### 3.2 Constant Laplacian smoothing

**Fig 1:**
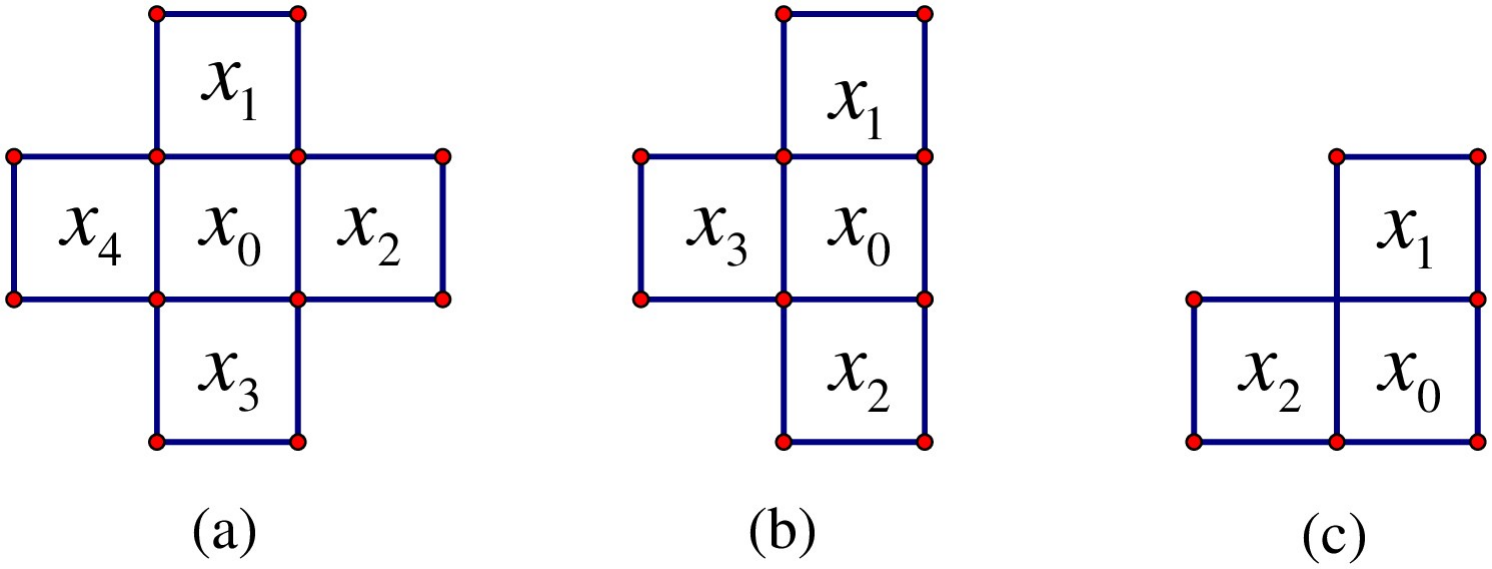
Three cases with Laplacian smoothing. (a) *x*_0_ is in the middle of the grid; (b) *x*_0_ is on the boundary of the grid except the corner; (c) *x*_0_ is in the corner of the grid.

Before introducing the new tomography algorithm, we will present Laplacian smoothing which is the basis of the new algorithm. Laplacian smoothing is an algorithm that can smooth polygon meshes based on temporal or spatial continuity. The smoothing operation is as follows: The value *x*_0_ of the voxel is replaced by the average of its neighboring voxels. For tomography rectangular grid, we only consider the nearest voxels. Because reconstruction region has strict boundaries, there are three cases when we use Laplacian smoothing, as shown in Fig 1.

In Fig 1, *x*_1_, *x*_2_, *x*_3_ and *x*_4_ are the nearest voxels to *x*_0_ in a CIT region. We use Laplacian smoothing method to constrain them. It can be expressed as

$$x_{1}+x_{2}+x_{3}+x_{4}-qx_{0}=0$$ (10)

in Fig 1(a),

$$x_{1}+x_{2}+x_{3}-qx_{0}=0$$ (11)

in Fig 1(b) and

$$x_{1}+x_{2}-qx_{0}=0$$ (12)

in Fig 1(c).

where *q* is smoothing factor whose initial values are 4, 3 and 2 for Fig 1(a), 1(b) and 1(c), respectively. The three equations are used to construct each voxel as constant smoothing constraints in the horizontal and vertical directions, respectively. However, constant smoothing factors may be inaccurate. Thus, we use an adaptive Laplacian smoothing method to reconstruct IED distribution.

### 3.3 Adaptive Laplacian smoothing

If unreasonable constraints are imposed, the accuracy of the IED reconstruction will be significantly reduced. To make the constraints closer to the actual situation, we develop an adaptive smoothing algorithm by interacting between constraints and reconstruction results. Firstly, we use the Laplacian smoothing to impose the initial horizontal and vertical constraints and use the ART algorithm to inverse the initial solutions. Then, we use the initial solutions to estimate the smoothing factors *q* with Eqs (10) to (12) and establish the new horizontal and vertical constraints. The new ionospheric tomography approach ALS-ART is performed as follows.

1. Use Eqs (10)–(12) to establish the initial horizontal and vertical constraints, respectively.
2. Calculate IED values by Eq (9).
3. Update the smoothing factors based on the new IED values from step (2).
  $$q=\{\begin{array}{cc} m & \text{if}\;x_{0}\leq x_{h}\\ \frac{\sum_{i=1}^{m}x_{i}}{x_{0}} & \text{if}\;x_{0}>x_{h} \end{array}$$ (13)
  where *m* is 4, 3 and 2 from Eqs (10) to (12), respectively. *x*_*h*_ is a threshold to prevent updating the smoothing factor from inaccurate results and is a half of the maximum IED in the results. We set a scale factor to update the *x*_*h*_ until it is less than triple root mean square error of IED. Repeat steps (2)-(3) until the difference between the IED values are smaller than the empirical 0.3 TECu.

## 4. Experimental results

**Fig 2:**
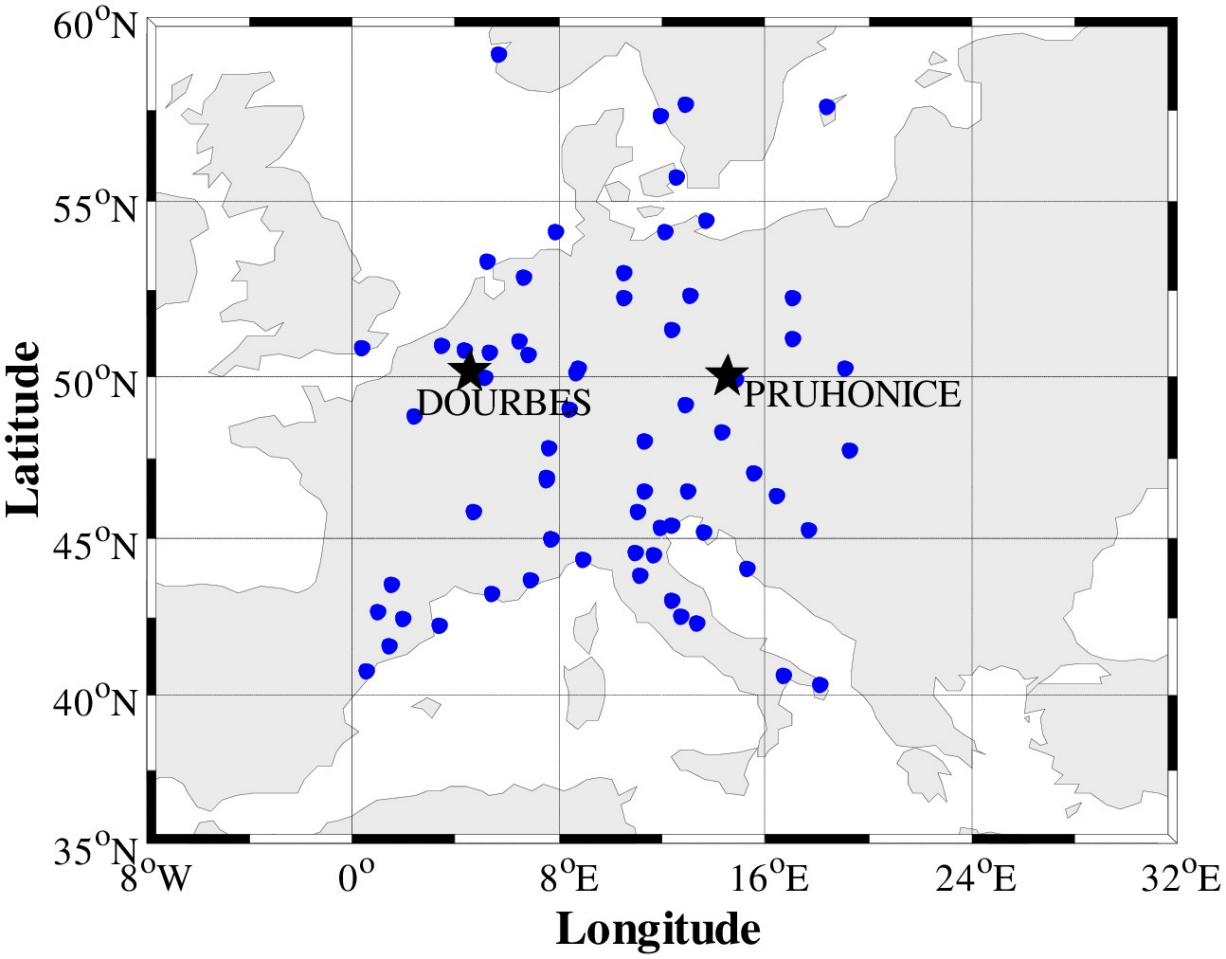
Distribution of GNSS observation and ionosonde stations.

To demonstrate the feasibility of the ALS-ART algorithm, some experiments are carried out with ALS-ART algorithm and constant Laplacian smoothing ART (CLS-ART) algorithm. The tomographic region extends from 0°E to 20°E in longitude, 40°N to 60°N in latitude, and 100 km to 1000 km in altitude. A spatial voxel is 1°×1°×50 km in the longitudinal, latitudinal and altitudinal directions. Thus, there are 8379 voxels in the three-dimensional grid. GNSS observations from the international GNSS service (IGS) stations in Europe are used in the experiments and consist of simulated observations and real measurements. Additionally, the data from ionosonde stations Dourbes (4.6°E, 50.1°N) and Pruhonice (14.6°E, 50.0°N) are used for independent testing and verification. The GNSS station distribution in the study region is shown in Fig 2 (“●” for GNSS stations and “★” for the ionosonde station).

All the data used in our experiments can be obtained as follows:

1. The GNSS data: we can obtain the data from the Crustal Dynamics Data Information System (CDDIS), and CDDIS supports data archiving and distribution activities for a global user community (ftp://cddis.gsfc.nasa.gov/pub/gps). Our data can be freely obtained by the URL: https://cddis.nasa.gov/archive/gps/data/daily/2018/238/18o.
2. The ionosonde data: NOAA’s National Centers for Environmental Information (NCEI) hosts and provides public access to users who archive for environmental data on Earth. The ionosonde data (Stations: DB049 and PQ052) in our experiment can be freely obtained from this organization by the URL: ftp://ngdc.noaa.gov/ionosonde/data/DB049/ and ftp://ngdc.noaa.gov/ionosonde/data/PQ052/.
3. The International Reference Ionosphere—IRI (2016): The Community Coordinated Modeling Center (CCMC) is a multi-agency partnership. The CCMC provides, to the international research community, access to modern space science simulations. In addition, the CCMC supports the transition to space weather operations of modern space research models. The IRI (2016) model in our experiment can be freely obtained by the URL: https://ccmc.gsfc.nasa.gov/modelweb/models/iri2016_vitmo.php.

### 4.1 Simulation

In this section, the real GNSS geometry used to construct matrix A in Eq (4) is used to calculate the slant TEC values for the simulated ionosphere. The simulated IED distributions are from 10:00 UT on 22 August 2018 and are provided by the international reference ionosphere (IRI) model. The simulated electron density volumes are taken from the IRI 2016 model. The observed random noise, which should follow a Gaussian distribution *ε* ~ *N*(0,0.01), is added to the simulated slant TEC values to obtain more realistic values that are used to test the efficiency of the method. To evaluate the accuracy of the reconstruction IED, the root mean square (RMS) error, average density error and maximum absolute error are used as the standards for CIT. The RMS error and average absolute error (AAE) can be respectively expressed as $RMS=\sqrt{\frac{1}{N}\sum_{j=1}^{N}{\left(Ne_{j}^{CIT}-Ne_{j}^{IRI}\right)}^{2}}$ and $AAE=\sum_{j=1}^{N}\left|Ne_{j}^{CIT}-Ne_{j}^{IRI}\right|/N$, where *N* is the total number of voxels in the inversion region, $Ne_{j}^{CIT}$ is the value of the IED reconstructed by CIT, and $Ne_{j}^{IRI}$ is the value of the IED obtained by IRI 2016.

**Fig 3:**
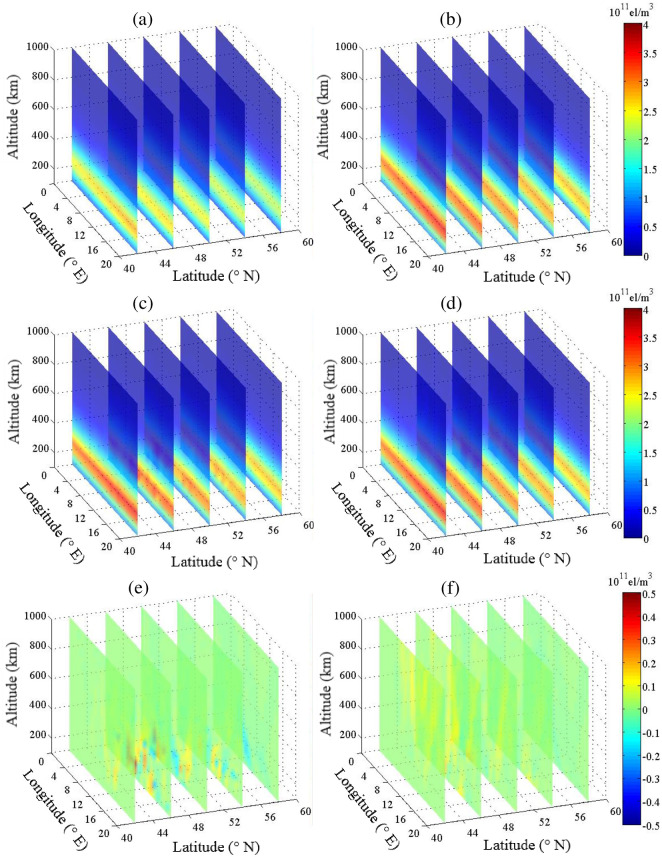
Estimated IED for CLS-ART (left panel) and ALS-ART (right panel) at various latitudes (unit: 10^11^ el/m^3^). (a) and (b) Background and simulated electron densities. (c) and (d) Estimated electron densities. (e) and (f) Error terms.

Fig 3 shows the estimated IED values for CLS-ART and ALS-ART at various latitudes. The IED and its error distribution reconstructed by CLS-ART are shown in Fig 3(c) and 3(e), respectively. The IED and its error distribution reconstructed by ALS-ART are shown in Fig 3(d) and 3(f), respectively.

**Fig 4:**
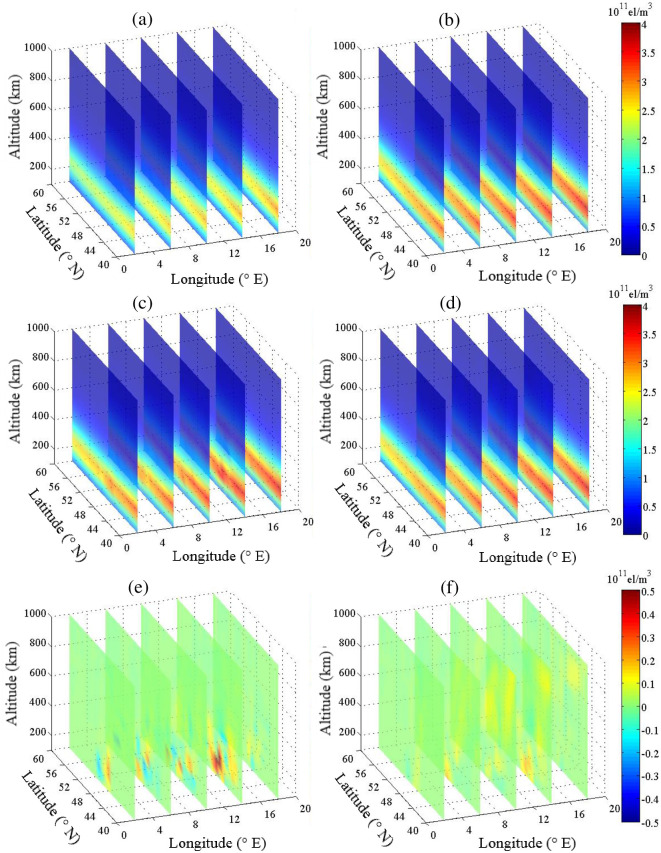
Estimated IED for CLS-ART (left panel) and ALS-ART (right panel) at various longitudes (unit: 10^11^ el/m^3^). (a) and (b) Background and simulated electron densities. (c) and (d) Estimated electron densities. (e) and (f) Error terms.

Fig 4 shows the estimated IED values for CLS-ART and ALS-ART at various longitudes. The IED and its error distribution reconstructed by CLS-ART are shown in Fig 4(c) and 4(e), respectively. The IED and its error distribution reconstructed by ALS-ART are shown in Fig 4(d) and 4(f), respectively. From the error terms in Figs 3 and 4, we can see that the accuracy of reconstructing IED using CLS-ART is obviously lower than that of reconstructing IED using ALS-ART between 100 km and 400 km.

Table 1 provides the reconstruction results of the error statistics for the two methods based on various longitudes. The maximum absolute error, average absolute error and RMS error of IED with the ALS-ART algorithm are smaller than those of IED with the CLS-ART algorithm at various longitudes.

**Table 1:** Error analysis of IED reconstruction using the two methods at various longitudes (unit: 10^10^ el/m^3^).

Longitude	ALS-ART			CLS-ART		
	Maximum absolute error	Average absolute error	RMS error	Maximum absolute error	Average absolute error	RMS error
2°E	0.84	0.06	0.12	2.58	0.07	0.28
6°E	2.11	0.16	0.19	3.09	0.16	0.31
10°E	1.45	0.28	0.36	4.11	0.24	0.40
14°E	2.16	0.24	0.28	5.64	0.30	0.62
18°E	0.99	0.13	0.19	1.36	0.09	0.13

**Fig 5:**
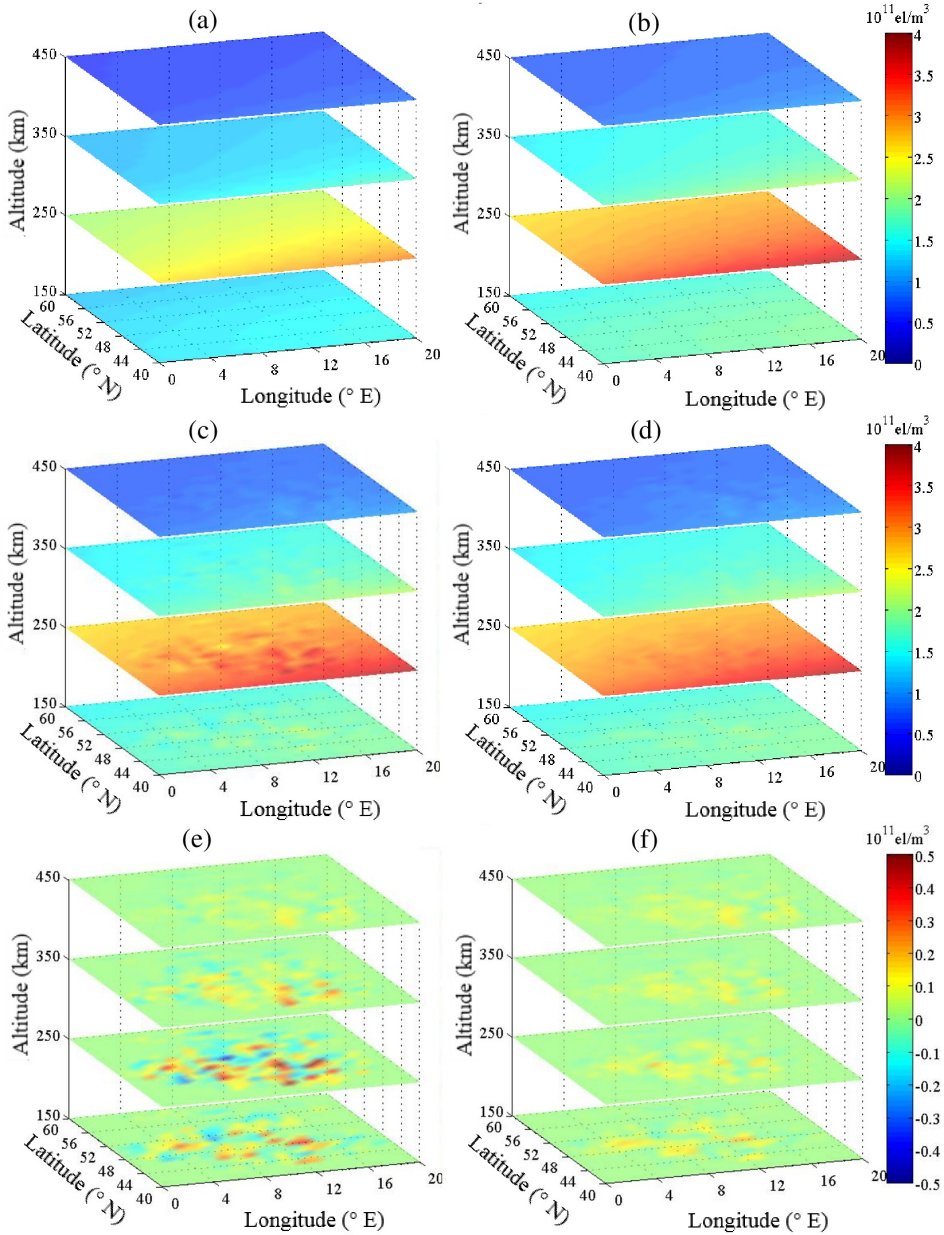
Estimated IED for CLS-ART (left panel) and ALS-ART (right panel) at various altitudes (unit: 10^11^ el/m^3^). (a) and (b) Background and simulated electron densities. (c) and (d) Estimated electron densities. (e) and (f) Error terms.

Fig 5 shows the estimated IED values for CLS-ART and ALS-ART at various Altitudes. The IED and its error distribution reconstructed by CLS-ART are shown in Fig 5(c) and 5(e), respectively. The IED and its error distribution reconstructed by ALS-ART are shown in Fig 5(d) and 5(f), respectively. From the error terms in Fig 5, we can see that the accuracy of reconstructing IED using CLS-ART is obviously lower than that of reconstructing IED using ALS-ART at 150 km, 250 km, 350 km and 450 km of altitude.

Table 2 represents the error analysis of IED reconstruction by using the two methods at various heights, and the results also presents the reconstruction results of the error statistics for the two methods based on various altitudes. The maximum absolute error, average absolute error and RMS error of IED with the ALS-ART algorithm are also smaller than those of IED with the CLS-ART algorithm at various longitudes. These results indicate that the CIT accuracy of the ALS-ART algorithm is generally superior to that of the CLS-ART algorithm. In addition, the maximum absolute error, average absolute error and RMS error of IED with the ALS-ART algorithm based on total voxels are approximately 3.51×10^10^ el/m^3^, 0.25×10^10^ el/m^3^ and 0.36×10^10^ el/m^3^, respectively. The maximum absolute error, average absolute error and RMS error of IED with the CLS-ART algorithm based on total voxels are approximately 6.73×10^10^ el/m^3^, 0.43×10^10^ el/m^3^ and 0.52×10^10^ el/m^3^, respectively. We believe that the ALS-ART algorithm has the validity and superiority. Additionally, we think that the ALS-ART algorithm can derive more reliable values and obtain results closer to the true values compared with the CLS-ART algorithm.

**Table 2:** Error analysis of IED reconstruction using the two methods at various heights (unit: 10^10^ el/m^3^).

Altitude	ALS-ART			CLS-ART		
	Maximum absolute error	Average absolute error	RMS error	Maximum absolute error	Average absolute error	RMS error
150 (km)	2.16	0.18	0.26	4.01	0.35	0.39
250 (km)	2.16	0.17	0.25	5.45	0.56	0.65
350 (km)	1.60	0.16	0.25	3.31	0.30	0.40
450 (km)	1.49	0.16	0.24	1.44	0.15	0.20

### 4.2 Real observation data

**Fig 6:**
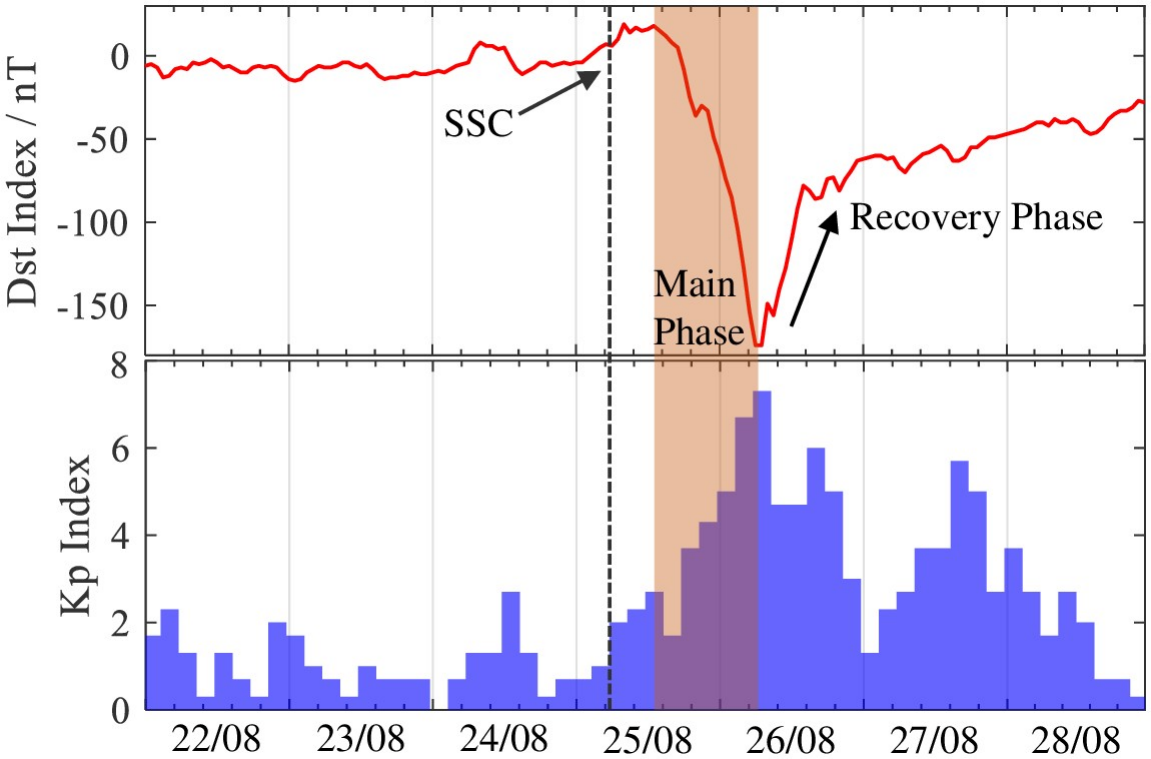
Temporal variations of Dst and Kp indexes during 22–28 August 2018.

Fig 6 shows that the Dst and Kp are used to analyse the possible influence of solar and geomagnetic index on the ionospheric disturbance responses. The Dst index suddenly turns northward at 06:00 UT and then rose to 19 nT at 08:00 UT on 25 August 2018. The geomagnetic storm sudden commencement (SSC) starts at 06:00 and lasted for 10 hours. The Dst index decreases rapidly at beginning of 17:00 UT on 25 August 2018, and reaches the minimum of -174 nT at 07:00 UT on 26 August 2018. The Kp index reaches the maximum of 7.3. In this section, we choose quiet day on August 22, 2018 and storm day on August 26, 2018 for experimental analysis.

We use observed slant TEC data from the IGS in Europe with a vertical profile derived from the two ionosonde stations. We use the real measurements from 22 August 2018, when the ionosphere was under calm conditions.

**Fig 7:**
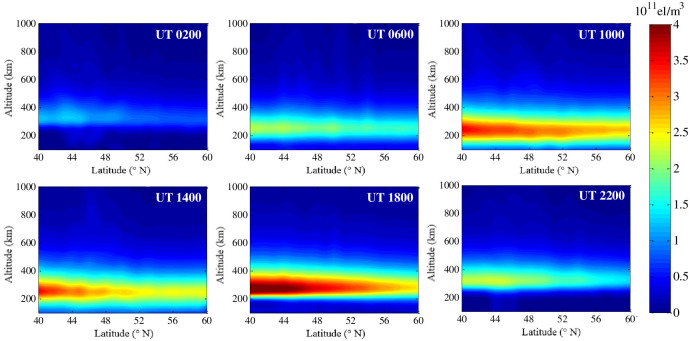
Tomographic images on 22 August 2018 with a fixed longitude meridian of 10°E.

Fig 7 demonstrates the hourly variation of the IED at a fixed longitude meridian of 10°E on 22 August 2018. This figure shows that the IED characteristics of the different latitudes are quite different, and the values of IED in the north is smaller than those of IED in the south as a whole. The characteristics indicate a strong correlation between IED and latitude. Comparing all the sub-graphs in Fig 7, it can be seen that the peak height of the IED gradually decreases in the period of 2:00 UT to 10:00 UT. Then, it gradually increases in the next time period. The peak height of the IED rises to 350 km at 22:00 UT, which presents the characteristics of the vertical variation of the IED this day.

**Fig 8:**
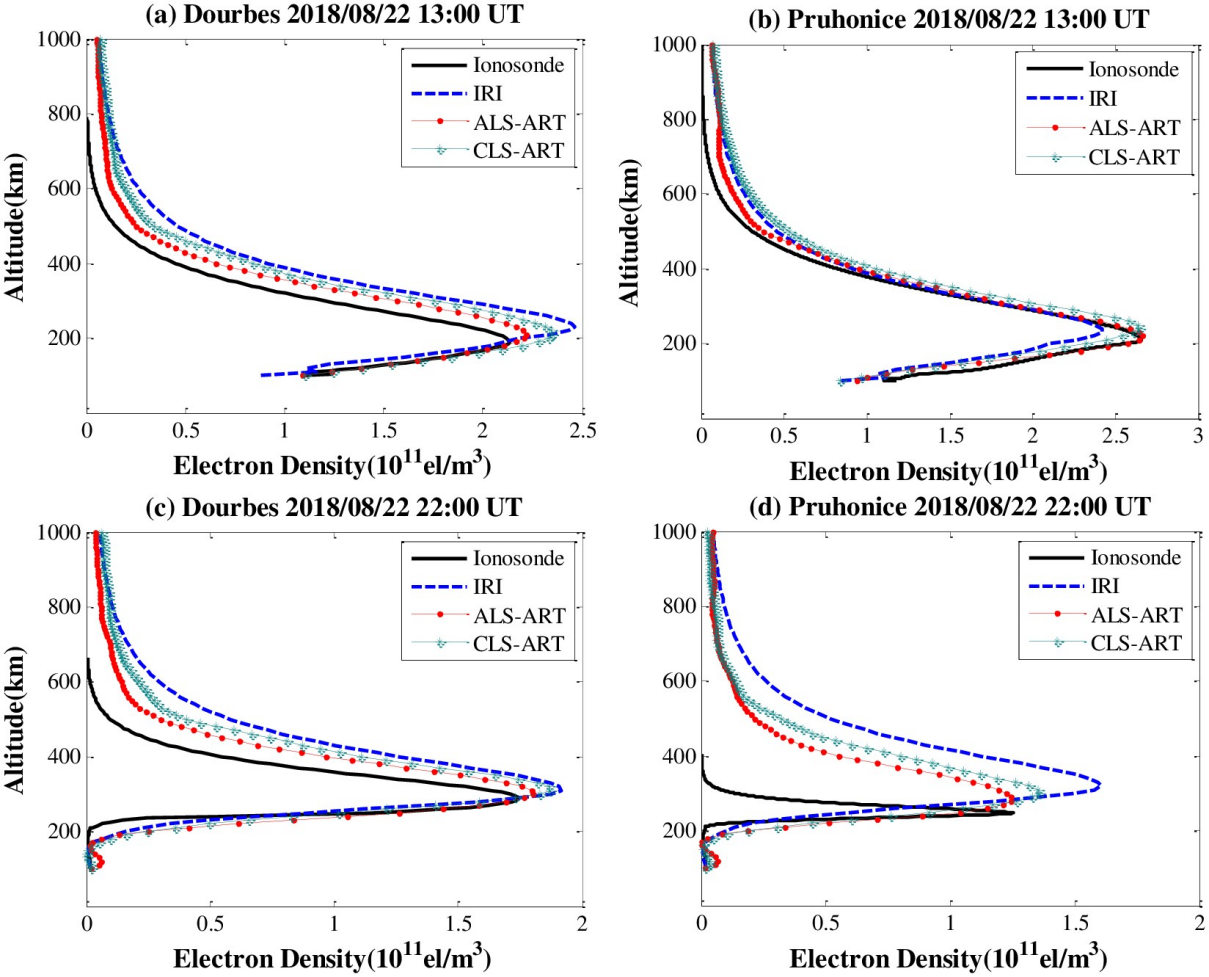
Comparison of the estimated IED profiles based on two algorithms with the IED profiles from the two ionosonde stations on 22 August 2018. (a) and (b) 13:00 UT. (c) and (d) 22:00 UT.

To verify the reliability of the CIT results, we use the ALS-ART algorithm to inverse IEDs which are compared with ionosonde data. Fig 8 shows the comparison of the profiles obtained by the Dourbes and Pruhonice ionosonde stations at different times with the inversion results of the two methods. Fig 8(a) and 8(b) are the comparison results of the electron density profiles at 13:00 UT. The average absolute error of IED with the ALS-ART algorithm based on Dourbes and Pruhonice are approximately 0.82×10^10^ el/m^3^ and 0.42×10^10^ el/m^3^, respectively. The maximum absolute error of IED with the CLS-ART algorithm based on Dourbes and Pruhonice are approximately 1.55×10^10^ el/m^3^ and 0.97×10^10^ el/m^3^, respectively. Fig 8(c) and 8(d) are the comparison results of the electron density profiles at 22:00 UT. The average absolute error of IED with the ALS-ART algorithm based on Dourbes and Pruhonice are approximately 0.63×10^10^ el/m^3^ and 1.33×10^10^ el/m^3^, respectively. The maximum absolute error of IED with the CLS-ART algorithm based on Dourbes and Pruhonice are approximately 0.86×10^10^ el/m^3^ and 1.59×10^10^ el/m^3^, respectively. In the figure, the inversion results of the ALS-ART algorithm are closer to the ionosonde measurements than those of the CLS-ART algorithm. This result shows that the inversion accuracy of the ALS-ART algorithm is better than that of the CLS-ART algorithm.

**Fig 9:**
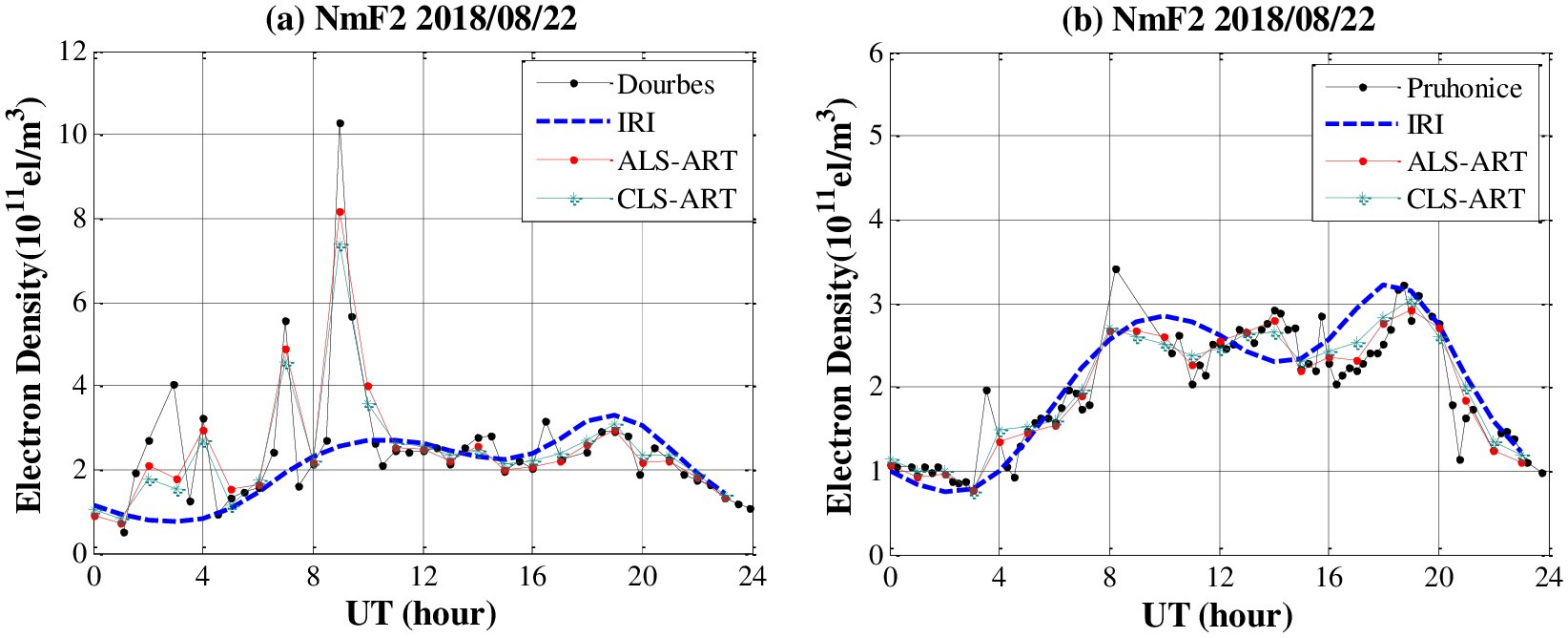
Comparison of the NmF2 values obtained from CIT and ionosonde data from the two ionosonde stations on 22 August 2018.

Fig 9 shows a comparison of the F2 layer peak electron density (NmF2) obtained from the two algorithms, as well as the observation data from the ionosonde stations at hourly time intervals on 22 August 2018. The peak in the electron density of the F2 layer obtained by two algorithms is generally in good agreement with that based on ionosonde measurements, but the peak obtained by the ALS-ART algorithm is generally closer to the ionosonde measurements than that based on CLS-ART algorithm.

For further validation, we give several examples of CIT results for regional IED distribution during the geomagnetic storm on 26 August 2018. The results are represent in Fig 9.

**Fig 10:**
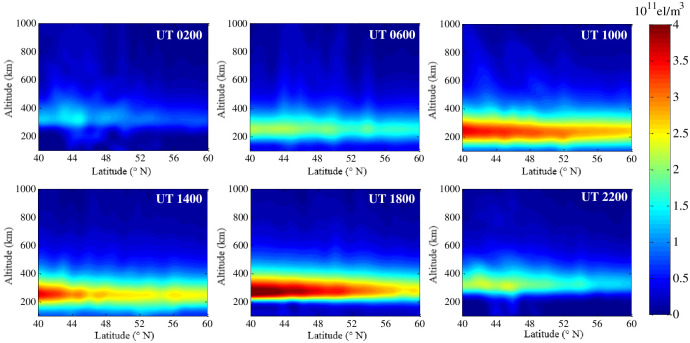
Tomographic images on 26 August 2018 with a fixed longitude meridian of 10°E.

Fig 10 demonstrates the hourly variation of the IED at a fixed longitude meridian of 10°E on this day. This figure also shows that the IED characteristics of the different latitudes are quite different, and the values of IED in the north is smaller than those of IED in the south as a whole. The peak height of the IED gradually decreases to 250 km in the period of 2:00 UT to 10:00 UT. Then, it gradually increases in the next time period. The peak height of the IED rises to 350 km at 22:00 UT, which also presents the characteristics of the vertical variation of the IED this day.

**Fig 11:**
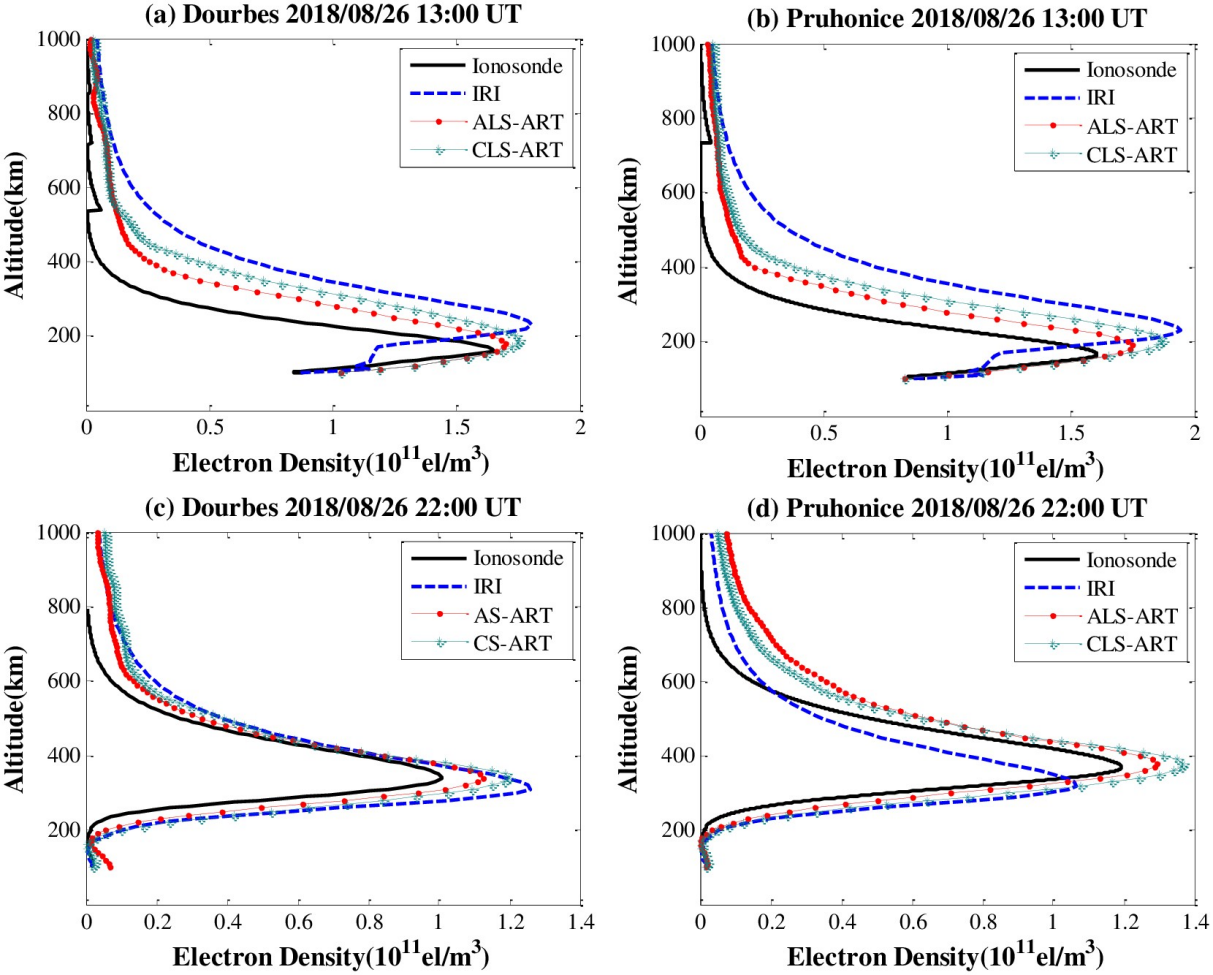
Comparison of the estimated IED profiles based on two algorithms with the IED profiles from the two ionosonde stations on 26 August 2018. (a) and (b) 13:00 UT. (c) and (d) 22:00 UT.

Fig 11 shows the IED profiles at two ionosode stations on 26 August 2018 at 13:00 UT and 22:00 UT. In general, the IED profiles derived from the ALS-ART algorithm match the ionosonde profile better than those derived from the CLS-ART algorithm. In Fig 11(a) and 11(b), the average absolute error of IED with the ALS-ART algorithm based on Dourbes and Pruhonice are approximately 1.23×10^10^ el/m^3^ and 1.07×10^10^ el/m^3^, respectively. The maximum absolute error of IED with the CLS-ART algorithm based on Dourbes and Pruhonice are approximately 1.51×10^10^ el/m^3^ and 1.29×10^10^ el/m^3^, respectively. In Fig 11(c) and 11(d), the average absolute error of IED with the ALS-ART algorithm based on Dourbes and Pruhonice are approximately 0.91×10^10^ el/m^3^ and 1.31×10^10^ el/m^3^, respectively. The maximum absolute error of IED with the CLS-ART algorithm based on Dourbes and Pruhonice are approximately 1.28×10^10^ el/m^3^ and 1.57×10^10^ el/m^3^, respectively. This result further validates that the ALS-ART algorithm is superior to the CLS-ART algorithm for the tomographic reconstruction of the IED under disturbed ionospheric conditions. An improvement in the NmF2 values is very obvious, but the peak height hmF2 values are apparently not well reconstructed. In Fig 11(a) and 11(b), the peak height based on the ALS-ART algorithm is slightly higher than that of the ionosonde data.

**Fig 12:**
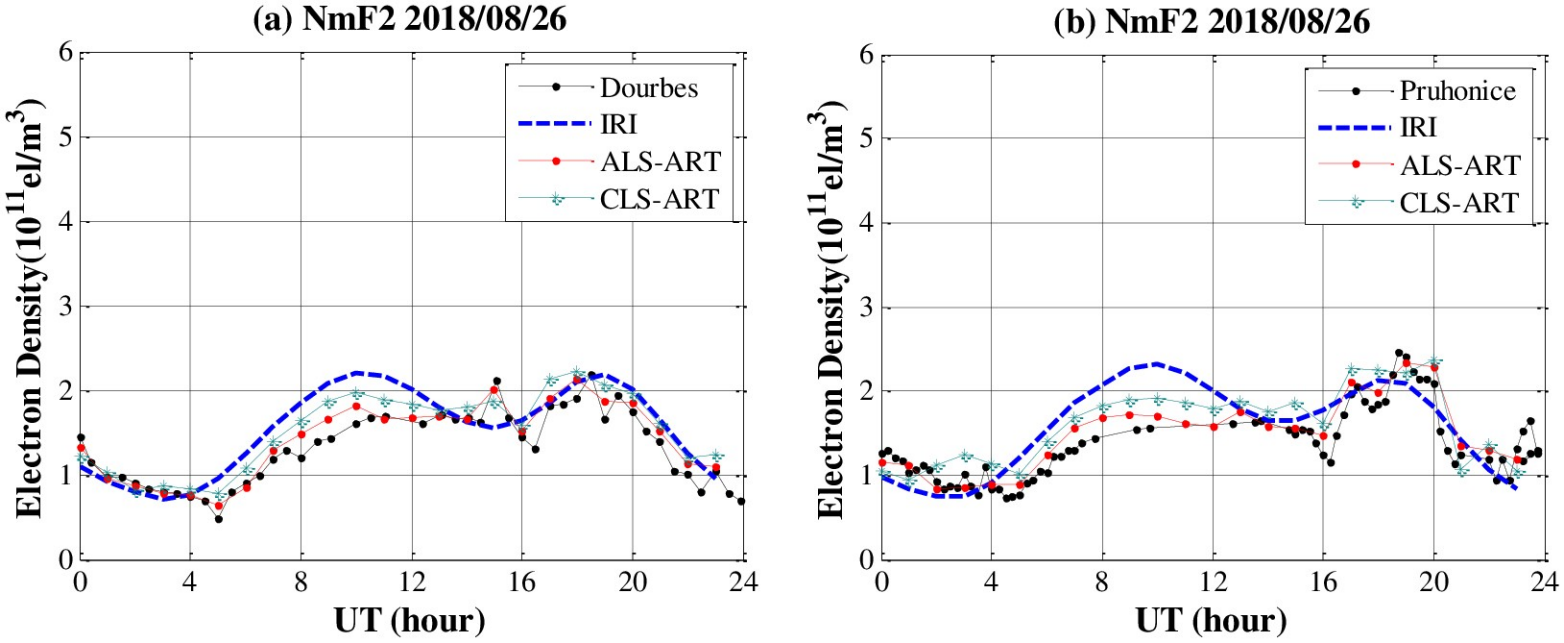
Comparison of the NmF2 values obtained from CIT and ionosonde data from the two ionosonde stations on 26 August 2018.

**Fig 13:**
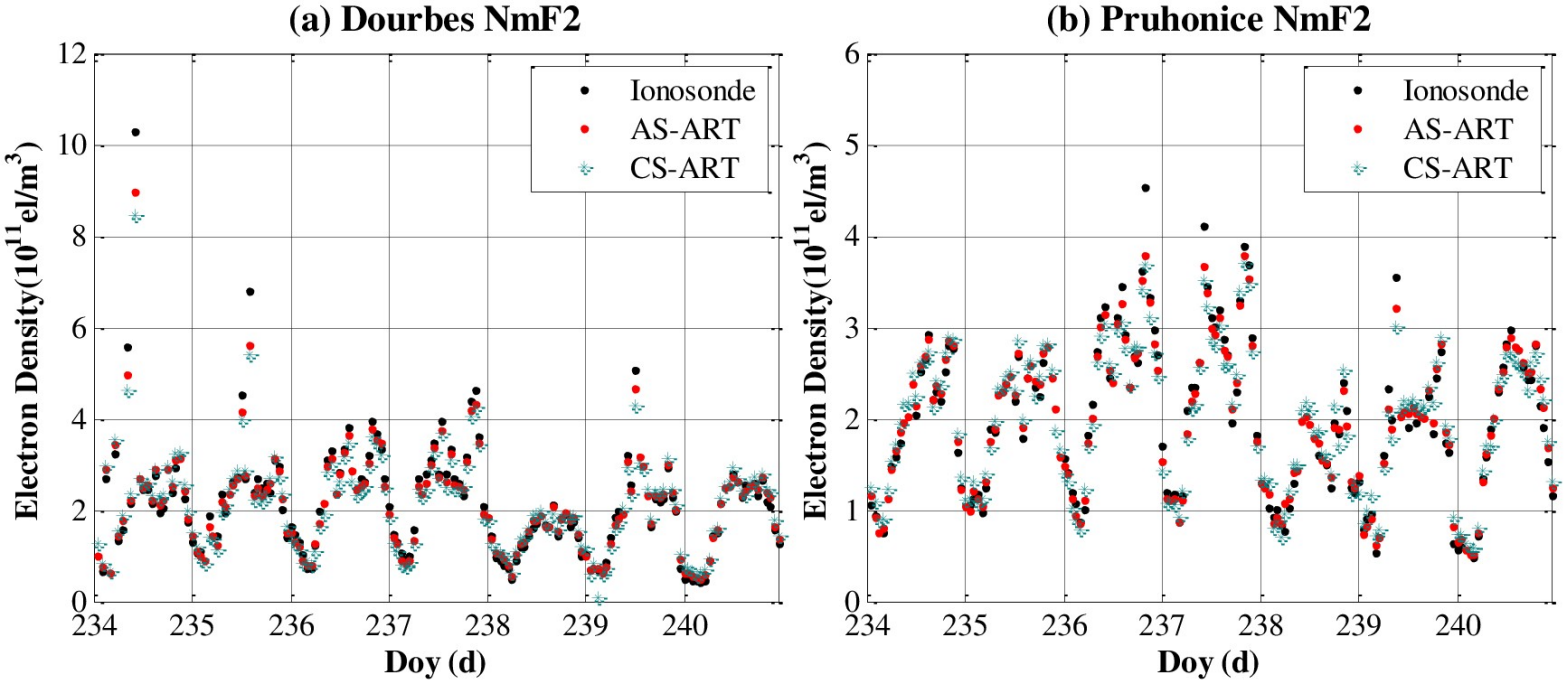
Comparison of NmF2 values obtained from CIT and ionosonde data from the two ionosonde stations from Doy 234 to 240 in 2018. (a) Dourbes. (b) Pruhonice.

Fig 12 shows the characteristics of the reconstructed F2 layer are validated against those of the ionosonde measurements. It is clear that the ALS-ART algorithm can improve the estimated NmF2 values with respect to the CLS-ART algorithm. Due to GNSS observation noise and station geometry limitations, the vertical resolution of the CIT results still needs to be improved. To evaluate reliability of the proposed method, we show the F2 layer peak density (NmF2) using the two methods with one week data. As shown in Fig 13, the characteristics of the reconstructed NmF2 are validated against those of the ionosonde measurements. Generally, the AS-ART method can improve the estimated NmF2 values with respect to the CS-ART method.

Reconstruction of tomographic images based on GNSS is a mathematical inversion problem. Iterative algorithms are commonly applied, but it is not well understood how good the solutions are. Depending on a prior information, the iteration may lead to different results which all satisfy the original measurements equally well [19]. The quality of the reconstruction results depends on how suitable the prior information are. The solution of smoothing constraints is well understood. It presents the most probable values of the unknowns by conditional constraints once the voxels without any observation information. One drawback of the smoothing operator is that, for applications in which IED spatial distribution contain discontinuities, the CLS-ART algorithm could present incorrect results. In the case of steep gradients, the adaptive smoothing operator should be used in order to allow for more variation between voxels. In this study, we have proposed a novel approach ALS-ART and tested the results from this algorithm inversion of CIT against independent ionosonde measurements. In general, the inversion accuracy of the ALS-ART algorithm is better than that of the CLS-ART algorithm. An improvement in the NmF2 values is very obvious. The inversion results of the method are different from the ionosonde measurements in the vertical direction because the grid should be unrefined in the vertical direction.

## 5. Conclusion

In this study, we implement the novel approach based on adaptive smoothing and algebraic reconstruction technique (ALS-ART) and conduct a series of ionospheric tomography experiments to investigate the accuracy of inversion results using the algorithm. The algorithm imposes adaptive constraints according to the smoothness of near voxels. To some extent, it overcomes the disadvantages of the CLS-ART algorithm. Experiments involving simulated values and real measurements are performed to validate the feasibility and superiority of the proposed approach compared to CLS-ART algorithm. The ALS-ART algorithm have been used to reconstruct IED under geomagnetic calm and storm conditions. The IED profiles inversed from ALS-ART match better with ionosondes than those from CLS-ART. Although the method improves the ionospheric tomography results, it cannot completely improve the vertical resolution of tomography. However, it is necessary to further add the quantity of observation information.
